# Ecosystem Resilience and Land-Use Governance: Remote Sensing Insights for Environmental Management in a Tropical Dry Forest

**DOI:** 10.1007/s00267-026-02629-4

**Published:** 2026-09-24

**Authors:** Lucas Nascimento da Silva, Bartolomeu Israel de Souza

**Affiliations:** Graduate Program in Development and Environment (PRODEMA), Federal University of Paraíba (UFPB), João Pessoa, Paraíba Brazil

**Keywords:** Caatinga, Net Primary Productivity, Governance regimes, Ecosystem resilience

## Abstract

Seasonally Dry Tropical Forests (SDTFs), such as the Brazilian Caatinga, are highly vulnerable to climate change and chronic anthropogenic disturbances. Despite extensive land-use conversion, understanding how vegetation productivity and resilience vary across different land governance regimes remains a critical gap. Here, we integrated two decades (2004–2024) of remote sensing data to evaluate Net Primary Productivity (NPP) and Water Use Efficiency (WUE). We applied interannual climate anomaly analyses (Z-scores) and adapted a trend-based classification framework to assess ecosystem trajectories. These biophysical metrics were cross-referenced with the spatial boundaries of Conservation Units, Indigenous Lands, Quilombola Territories, and private properties, including a distance-decay edge effect analysis. While long-term temporal trends for NPP and WUE remained relatively stable over the two decades, standardized anomaly analyses revealed a strong, statistically significant inverse coupling between productivity and water-use efficiency (*r* = − 0.741, *p* < 0.001), primarily mediated by surface moisture retention (LSWI) rather than immediate precipitation alone. Spatial analyses demonstrated that Conservation Units act as the main ecological refugia, maintaining peak restoration within a 0–2 km internal buffer. Notably, Indigenous Lands and Quilombola Territories exhibited remarkable edge-to-core resilience, maintaining homogeneous conservation levels that effectively buffer external degradation. To safeguard the Caatinga’s structural integrity against desertification, public policies must move beyond reactive drought subsidies towards proactive landscape management that legally empowers traditional communities and incentivizes conservation on private lands.

## Introduction

Seasonally Dry Tropical Forests (SDTFs) are extensive biomes with high biodiversity, yet they remain globally underprotected and increasingly threatened (Blackie et al. 2014; Dryflor et al. 2016; Miles et al. 2006). Despite playing a critical role in the terrestrial carbon cycle and in maintaining biodiversity under water-stressed conditions, SDTFs have experienced significant habitat loss over the years, leaving much of these biomes fragmented and increasingly pressured by agricultural activities and climatic variability (Antongiovanni et al. 2020; Pennington et al. 2009). Unlike humid forests, which historically have attracted greater attention from scientific and political agendas, dry forests are often overlooked in conservation efforts, even though they sustain water and food security for millions of people, particularly in developing countries (Faggin et al. 2017; Siegmund-Schultze, 2021). In the Anthropocene, where extreme climatic events are becoming more frequent, discussions on the functional stability of these ecosystems must be addressed urgently within the context of global environmental security (Huang et al. 2016; IPCC, 2023; Marengo et al. 2017).

Located in South America, the Caatinga stands out as the largest and most populous SDTF in the world, covering approximately 844,453 km^2^ in the Brazilian semi-arid region (Silva et al. 2017). Multiple studies and models indicate trends of aridification and increasing surface temperature in the region by the end of the century (Buriti & Barbosa, 2018; Marengo et al. 2017; Torres et al. 2017), positioning the Caatinga at the center of a multifaceted crisis and marking it as a climate change hotspot. In addition to climate change impacts, the Caatinga is also subject to chronic anthropogenic disturbances, such as deforestation and soil degradation, where biomass exploitation and landscape fragmentation reduce ecosystem resilience and accelerate desertification processes (Mariano et al. 2018; Tomasella et al. 2018; Vieira et al. 2015). Recent land-use and land-cover assessments quantify the extent of this transformation: between 1985 and 2019, Forest Formation and Savanna Formation areas within the Caatinga declined by approximately 8% and 11%, respectively, while pastureland expanded by 62% and agricultural land by 284% (Rocha et al. 2024), underscoring the scale of vegetation conversion underlying the biome’s structural fragmentation.

Despite these severe threats and historical neglect, there remains a gap in understanding how vegetation productivity responds to emerging climate scenarios. Among the vegetation metrics available from remote sensing (e.g., NDVI, EVI, GPP), NPP was selected because it represents the ecosystem’s net carbon gain after autotrophic respiration, integrating photosynthetic activity and thermal-hydric constraints into a single, ecologically interpretable proxy for the biome’s carbon sequestration capacity and functional health (Potter et al. 1993). Traditionally, NPP in drylands has been suggested to be linearly coupled with annual precipitation (Ma et al. 2023). However, recent evidence indicates a nonlinear dynamic, in which Water Use Efficiency (WUE)—the ratio between carbon assimilated through NPP and water lost via evapotranspiration, and therefore a direct measure of the ecosystem’s physiological efficiency in converting scarce water into biomass—and foliar phenology can be decoupled from immediate rainfall pulses, depending on the soil-plant system’s moisture retention capacity (Costa et al. 2022; Medeiros et al. 2022; Mendes et al. 2025). Because WUE captures the trade-off between productivity gains and water cost, it offers land managers an ecologically meaningful indicator for anticipating vegetation vulnerability and prioritizing soil-water conservation interventions, complementing what precipitation or NPP trends alone can reveal. The use of water-sensitive indices, such as LSWI, and temperature-sensitive indices, such as TVDI, integrated into process-based models like CASA (Carnegie-Ames-Stanford Approach), has revealed that ecological memory plays an underestimated role in maintaining ecosystem carbon stocks (Bao et al. 2016; Rodigheri et al. 2024; Xing et al. 2024; Zhang et al. 2023). Recent studies confirm that the Caatinga can function as an extremely efficient carbon sink in years with favorable environmental conditions, challenging the traditional view of low productivity in dry forests (Castanho et al. 2020; Mendes et al. 2025; Morais et al. 2017).

In addition to biophysical variables, the resilience of the Caatinga is intrinsically influenced by land governance. The region’s landscape is commonly composed of mosaics of tenure regimes, including private properties, Protected Areas (PAs), Indigenous Lands, and Quilombola Territories. While some global studies focus on the effectiveness of state-managed parks (Oliveira et al. 2022; Teixeira et al. 2021), there remains a critical lack of quantitative assessments on the role of traditional communities in maintaining ecosystem functionality in dry forests (Dawson et al. 2023; Freire et al. 2020; Sangat et al. 2025). Recent research suggests that Afro-descendant and Indigenous Territories, as well as Private Natural Heritage Reserves (RPPNs), can act as vital barriers against degradation, often surpassing the effectiveness of strictly protected areas under low implementation (Benfica et al. 2022; Nascibem et al. 2023; Souza et al. 2025). Beyond their internal conservation performance, protected and traditional territories may also shape the surrounding landscape through edge effects: agricultural encroachment and resource extraction can degrade functionality near territorial boundaries (spillover), or, conversely, well-enforced boundaries may buffer core areas from these external pressures, isolating them from adjacent land-use change (Antongiovanni et al. 2020; Dawson et al. 2023). Determining which of these dynamics predominates in the Caatinga has direct implications for the design of buffer zones and ecological corridors around protected and traditional territories.

In this context, the present study aims to fill these gaps by integrating remote sensing time series (2004–2024) with advanced ecological modeling on the Google Earth Engine platform (Chen et al. 2025; Gorelick et al. 2017; Tesfaye et al. 2024) and programming tools. This research adopts a functional perspective at the biome scale, aiming to: (i) estimate NPP and WUE using an adapted CASA model; (ii) identify climatic vectors driving recent changes through statistical analyses (Li et al. 2022; Qi et al. 2023); and (iii) compare the effectiveness of different governance regimes (PAs, Indigenous, and Quilombola territories) in maintaining ecosystem resilience in the Caatinga.

## Materials and Methods

### Study Area

**Fig. 1:**
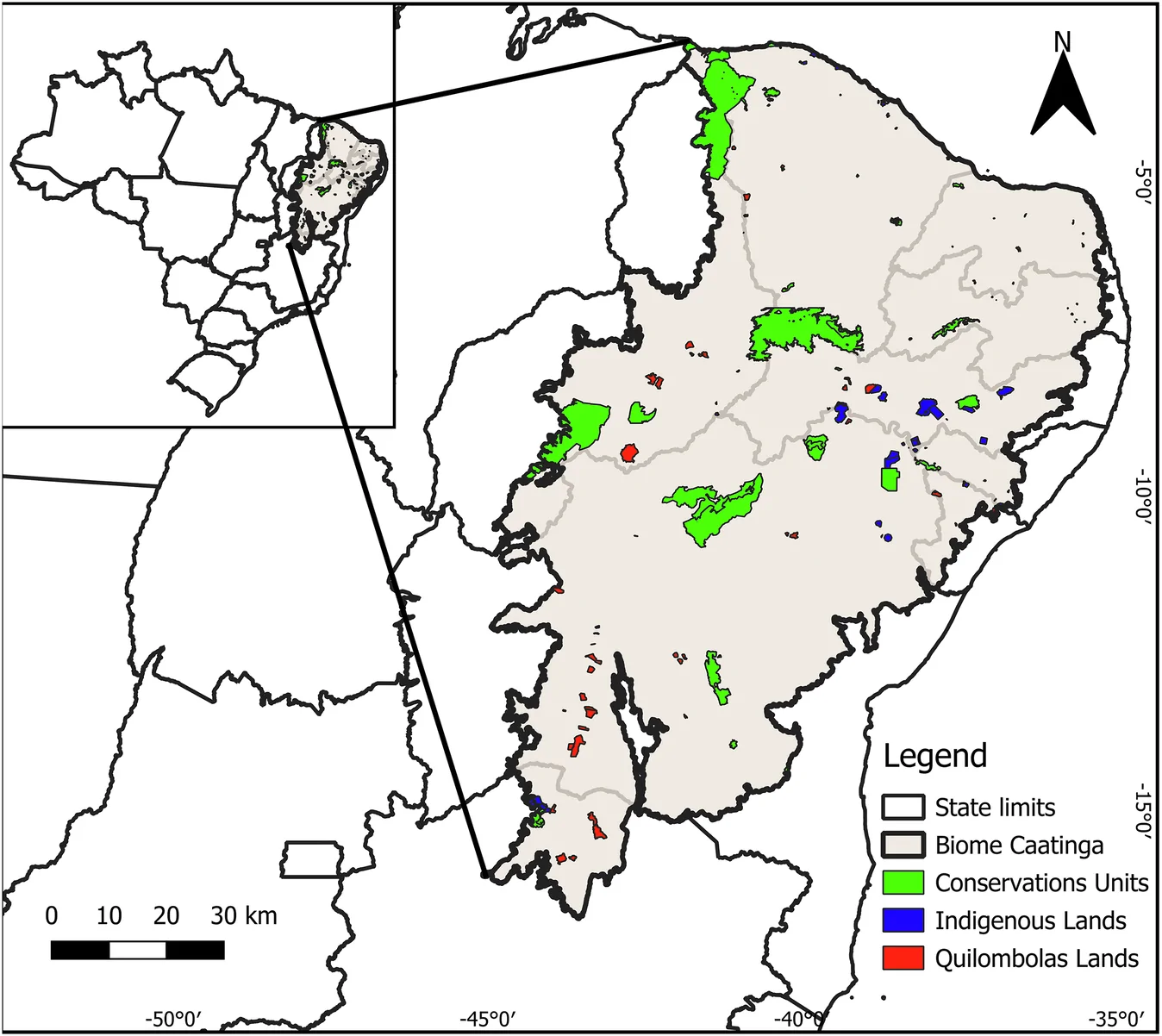
Location map of the Caatinga biome and distribution of governance regimes

This study encompassed the entire Caatinga biome, located in northeastern Brazil (Fig. 1), covering approximately 10% of the national territory. This biome is predominantly characterized by a semiarid climate, with high mean annual temperatures (25–30 ^∘^C) and irregular precipitation patterns, ranging from 300 to 800 mm per year, mostly concentrated within a 3–4 months period, resulting in dry periods that can extend up to 8 months (Silva et al. 2017).

The vegetation is primarily composed of a heterogeneous mosaic of deciduous dry forests, shrublands, and herbaceous formations, all adapted to this strong seasonal precipitation regime. The biome’s phenological dynamics are deeply coupled with water availability. The onset of the short rainy season triggers a rapid leaf flush that quickly restores the canopy and peaks ecosystem productivity, whereas during the prolonged dry season, the vegetation undergoes extensive leaf senescence as a physiological mechanism to minimize water loss (Costa et al. 2022; Medeiros et al. 2022; Mendes et al. 2025). Despite these harsh environmental constraints, the Caatinga harbors exceptional biodiversity and can function as a highly efficient carbon sink in years with favorable conditions, challenging the traditional view of low productivity in dry forests (Mendes et al. 2025; Morais et al. 2017).

### Data and Methodological Workflow

**Fig. 2:**
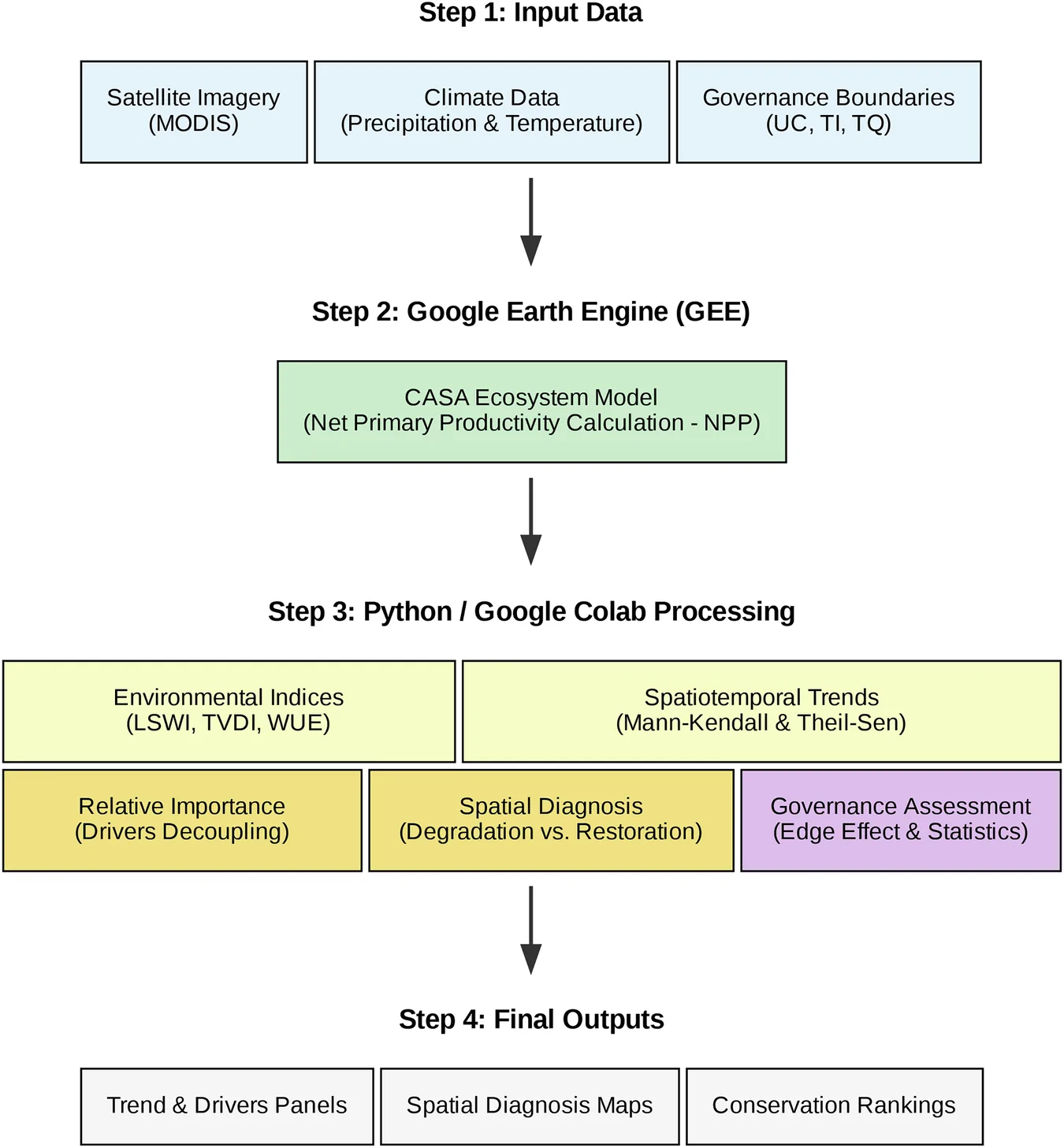
General flowchart of the methodological process, from data acquisition to results analysis

The workflow adopted in this study integrated the processing of large volumes of spatial data with biophysical and spatial statistical modeling, as illustrated in the general flowchart (Fig. 2).

Time series spanning the period 2004–2024 were used, derived from orbital sensors and reanalysis models. Temporal consistency was prioritized, utilizing products from MODIS (collection 6.1), CHIRPS (precipitation), and ERA5-Land meteorological data. Monthly outputs from the CASA model, together with the climatic and spectral indices were aggregated into annual means, which constitute the sampling unit used throughout the trend analyses. Annual aggregation was adopted over sub-annual or dry-season-specific metrics because the study’s objective was to characterize the biome’s multi-annual functional trajectory; this choice smooths intra-annual variability, which future work could address through seasonal decomposition.

### Optimized CASA Model

The CASA model (Potter et al. 1993) was used to estimate Net Primary Productivity (NPP). Monthly NPP was calculated as the product of Absorbed Photosynthetically Active Radiation (APAR) and light-use efficiency (*ε*) (Eq. (1)).

1 $$NPP(x,t)=APAR(x,t)\times \varepsilon (x,t)$$

where *x* represents the pixel and *t* denotes time. For the APAR calculation, solar radiation (SOL) and the fraction of photosynthetically active radiation (FPAR) were combined (Eq. (2)).

2 $$APAR(x,t)=SOL(x,t)\times 0.5\times FPAR(x,t)$$

To mitigate signal saturation in areas of dense vegetation as well as soil noise in degraded areas, FPAR was calculated as the linear average of FPAR derived from NDVI and the Simple Ratio (SR) (Bao et al. 2016; Xing et al. 2024), as shown in Eq. (3):

3 $$FPAR(x,t)=\frac{FPA{R}_{NDVI}(x,t)+FPA{R}_{SR}(x,t)}{2}$$

Light-use efficiency (*ε*) was dynamically modeled and downregulated by temperature and water stress scalars (Eq. (4)):

4 $$\varepsilon (x,t)={\varepsilon }_{max}\times {T}_{1}(x,t)\times {T}_{2}(x,t)\times {W}_{\varepsilon }(x,t)$$

The *ε*_*max*_ represents the maximum theoretical efficiency possible for each vegetation type under optimal conditions. The detailed mathematical formulations for calculating the individual FPAR components (*FPAR*_*NDVI*_ and *FPAR*_*SR*_), as well as the thermal (*T*_1_, *T*_2_) and water (*W*_*ε*_) stress coefficients, are fully described in Appendix A.

Direct field validation using eddy covariance flux towers was not feasible for this study: although two towers have operated within the Caatinga, they cover a single pixel footprint and only overlapped in 2014–2015, providing insufficient spatial and temporal coverage for a biome-wide, two-decade assessment. To address this limitation, we adopted a cross-validation approach against consolidated global products, a strategy widely supported in regional-scale studies where field data are insufficient (Jiao et al. 2018; Turner et al. 2005). We therefore validated CASA-derived NPP against the annual accumulated estimates from the MOD17A3HGF product (*R*^2^ = 0.79; RMSE and bias reported in Supplementary Fig. S1). We acknowledge that this constitutes a model-to-model comparison rather than a fully independent validation, since both products share methodological assumptions rooted in light-use-efficiency theory; agreement between them should therefore be interpreted as internal consistency rather than absolute accuracy. Complementary carbon-accounting datasets, such as SEEG, Climate TRACE, and the MapBiomas Soil Carbon collection, could provide independent benchmarks in future work, though they operate at different spatial and temporal resolutions and were not incorporated here. The CASA model was retained because it enables methodological continuity with prior biome-scale assessments and provides an operational framework that can be progressively refined as field monitoring networks expand.

### Water Use Efficiency (WUE)

Ecosystem-level WUE was calculated as the ratio between carbon assimilation, here approximated by NPP (gC m^−2^ yr^−1^), and water loss through evapotranspiration (ET), obtained from the MOD16A2GF V6.1 product (500 m spatial resolution, 8-day composite, aggregated to annual totals in mm yr^−1^; NASA LP DAAC; Supplementary Table S1), over the same time interval (Costa et al. 2022; Tesfaye et al. 2024) (Eq. (5)). WUE is thus expressed in gC mm^−1^. This formulation allows for distinguishing whether increases in vegetation biomass are driven by greater water availability or by physiological adaptations of the local vegetation.

5 $$WUE=\frac{NPP}{ET}$$

### Climatic Variables and Stress Indices

To analyze the drivers of vegetation and land-use change, four main variables were processed: precipitation (P), temperature (T), and two spectral indices, LSWI (Land Surface Water Index) and TVDI (Temperature-Vegetation Dryness Index). LSWI is calculated from the near-infrared (NIR) and shortwave infrared (SWIR) bands, acting as a proxy for vegetation water content (Eq. (6)).

6 $$LSWI=\frac{{\rho }_{NIR}-{\rho }_{SWIR}}{{\rho }_{NIR}+{\rho }_{SWIR}}$$

TVDI, in turn, is an index that integrates land surface temperature (LST) and NDVI, and is sensitive to soil moisture conditions (Rodigheri et al. 2024) (Eq. (7)).

7 $$TVDI=\frac{LST-LS{T}_{min}}{LS{T}_{max}-LS{T}_{min}}$$

where *LST* is the observed temperature at the pixel; *LST*_*min*_ corresponds to the “wet edge” (maximum potential evapotranspiration, unlimited water availability) and *LST*_*max*_ to the “dry edge” (no water availability). These limits can be determined empirically for each monthly scene through linear regression of extreme pixels in the scatter space (Eqs. (8) and (9)).

8 $$LS{T}_{max}={a}_{dry}+{b}_{dry}\times NDVI$$

9 $$LS{T}_{min}={a}_{wet}+{b}_{wet}\times NDVI$$

TVDI values range from 0 (wet) to 1 (dry). This method is particularly effective in heterogeneous regions such as the Caatinga, as it normalizes the effect of vegetation cover on surface temperature (Ma et al. 2023; Rodigheri et al. 2024).

### Trend Analysis Using Theil-Sen and Mann-Kendall Tests

The detection of changes and trends in the time series was performed on a pixel-by-pixel basis. The Theil-Sen slope estimator was used to quantify the magnitude of annual change, owing to its robustness to outliers in nonparametric series. The statistical significance of trends was assessed using the Mann-Kendall test, distinguishing between significant and non-significant changes (Li et al. 2022; Ma et al. 2023) (Eq. (10)). To evaluate long-term temporal dynamics, trend analyses were applied to the annual averages of NPP, WUE, and climatic variables (Precipitation, Temperature, LSWI, TVDI). All indices were systematically analyzed using the exact same temporal trend framework (Theil-Sen and Mann-Kendall).

10 $$\beta ={{{\rm{Median}}}}\left(\frac{{x}_{j}-{x}_{i}}{j-i}\right),\quad \forall j > i$$

Where *β* represents the Theil-Sen slope and significance is given by the Mann-Kendall *Z* statistic.

The use of annual means was preferred over seasonal metrics to capture the net ecosystem response over complete hydrological cycles, effectively mitigating short-term seasonal noise typical of the Caatinga. Additionally, to investigate extreme droughts and anomalous ecosystem responses, a standardized climate anomaly analysis (Z-score) was incorporated. The Z-score was calculated for each variable by subtracting the historical mean of the series (2004–2024) from the annual value and dividing the result by the standard deviation. This allowed for the identification of extreme climatic deviations and their direct coupling with productivity drops or efficiency peaks.

Finally, Pearson correlation coefficients (*r*) and their respective *p*-values were applied to these standardized anomalies to quantify the strength and direction of the short-term linear coupling between vegetation metrics (NPP, WUE) and environmental drivers (LSWI, Precipitation), a parametric approach widely supported for evaluating ecohydrological responses in the Caatinga (Medeiros et al. 2022).

### Classification Framework

To characterize landscape change processes, we adapted the classification framework proposed by Zhang et al. (2023), which combines trend direction (Theil-Sen/Mann-Kendall) with a Hurst exponent-based persistence criterion. In this study, we retained the trend-based classification of NPP and WUE trajectories but did not compute the Hurst exponent, since reliable pixel-level estimation of long-range persistence typically requires longer and denser time series than the 21 annual observations available here (2004–2024). Pixels were therefore categorized into five functional classes based on trend significance and direction alone (Table 1). Because Zhang et al. (2023) developed their original framework for an arid oasis–desert system in which immediate precipitation pulses linearly dictate vegetation dynamics—whereas in the Caatinga, productivity is non-linearly mediated by the soil-plant system’s moisture retention capacity (as evidenced by the dominance of LSWI in our study)—comparisons between the two studies’ class proportions should be interpreted as illustrative rather than directly transferable. This adapted approach still allows differentiation between areas experiencing true greening and those exhibiting only a short-term response to rainfall.

**Table 1:** Classification of vegetation dynamics processes based on the combination of temporal trends of Net Primary Productivity (NPP) and Water Use Efficiency (WUE), adapted from Zhang et al. (2023)

Functional Class	NPP	WUE	Ecological Interpretation
Early Restoration	+	+	Simultaneous increases in biomass and resource-use efficiency, indicating healthy ecosystem recovery.
Late Restoration	+	−	Increase in biomass associated with higher water cost.
Early Degradation	−	+	Decline in biomass accompanied by increased efficiency, reflecting a survival response to severe drought stress.
Late Degradation	−	−	Functional collapse with simultaneous losses in productivity and water regulation capacity.
No Change	NS	NS	Non-significant trends (*p*≥0.05).

( + ) Positive trend; ( − ) Negative trend; *NS* Non-Significant.

### Governance and Edge Effect

The influence of governance regimes on the conservation of ecosystem functionality was assessed using zonal statistics. We used the most recent official vector data to delineate protected and recognized territories:

- **Conservation Units (CUs):** Data from the National Register of Conservation Units (CNUC/MMA, 2025 release), subdivided into Strict Protection and Sustainable Use.
- **Indigenous Lands (ILs):** Official polygons provided by the National Foundation of Indigenous Peoples (FUNAI, 2024 release).
- **Quilombola Territories (QT):** Titled areas provided by the National Institute for Colonization and Agrarian Reform (INCRA, 2024 release).
- **Other Areas (Non-Protected):** Remaining Caatinga areas not under official protection, used as a baseline for comparison.

NPP and the classification framework were intersected with the official boundaries of Conservation Units, Indigenous Lands, and Quilombola Territories. Additionally, we performed buffer analyses to examine degradation or regeneration gradients at distances of 0–2 km, 2–5 km, and 5–10 km from protected area boundaries, testing the hypothesis of spillover or isolation of conserved fragments (Antongiovanni et al. 2020; Dawson et al. 2023).

### Drivers Ranking Using Random Forest

To understand the complex and non-linear relationships between vegetation dynamics and environmental factors, we employed a Random Forest (RF) machine learning algorithm (Breiman, 2001). It is important to emphasize that while the RF model does not establish strict mechanistic causality, it is highly robust for quantifying the predictive power and hierarchical importance of different drivers in explaining the variance of a target variable.

The RF model was constructed using an ensemble of decision trees (e.g., *n*_*trees*_ = 500) to prevent overfitting. The model was trained using the temporal trends of NPP (and subsequently WUE) as the continuous target variables. A total of five primary predictors were included in the analysis: four continuous environmental metrics (Precipitation, Temperature, LSWI, and TVDI) and one categorical variable representing the land-use governance regimes (Conservation Units, Indigenous Lands, Quilombola Territories, and Unprotected Areas).

During the training process, each decision tree was built using an independent bootstrap sample of the data, and at each node, a random subset of predictor variables was evaluated to determine the optimal split. The relative importance of each driver was assessed by calculating the percentage increase in Mean Squared Error (%IncMSE) when the values of a given predictor were randomly permuted in the Out-of-Bag (OOB) sample. Variables with higher %IncMSE values are considered more critical to the model’s accuracy, enabling the reliable identification of the primary factors limiting or boosting vegetation productivity and water use efficiency across the Caatinga biome.

## Results

### Temporal Trends and Ecosystem Response

**Fig. 3:**
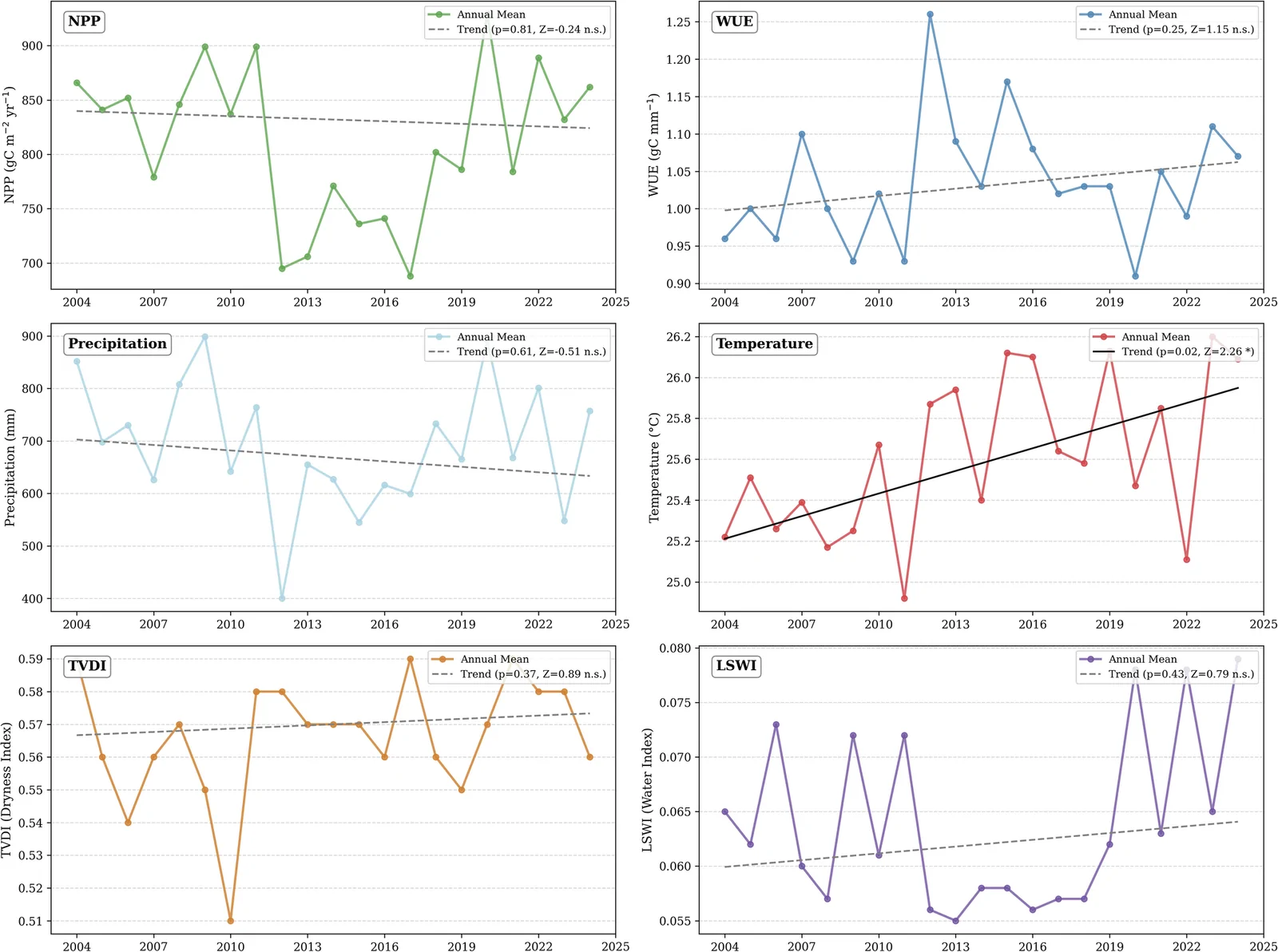
Interannual time series and trends (2004–2024) of NPP, WUE, precipitation, temperature, TVDI, and LSWI. The Mann-Kendall *Z* statistic is displayed in each panel alongside the fitted trend line. Non-significant trends (*p*≥0.05) are denoted by ‘n.s.’

Analysis of the interannual time series revealed stability in Net Primary Productivity (NPP) and Water Use Efficiency (WUE) in the Caatinga. As shown in Fig. 3, the NPP trend did not reach statistical significance (*p* = 0.803), with annual means fluctuating around the historical baseline. A similar stationary pattern was observed for WUE (*p* = 0.405), indicating that the balance between carbon fixation and water loss remained resilient over the past two decades. However, despite the absence of a monotonic long-term trend, the detrended anomaly analyses (Z-scores) revealed that interannual variations in NPP and WUE were highly responsive to short-term climatic fluctuations (see correlation heatmap in Supplementary Fig.e S2). Specifically, the standardized anomalies demonstrated a strong and statistically significant inverse coupling between productivity and water-use efficiency (*r* = − 0.741, *p* < 0.001), alongside a significant positive correlation between surface moisture (LSWI) and annual precipitation pulses (*r* = 0.630, *p* = 0.002). This statistical evidence explains why the biome exhibits extreme interannual volatility—functioning as a highly efficient carbon sink in wet years and experiencing severe productivity drops, with compensatory efficiency spikes, during droughts—without necessarily establishing a permanent directional trend.

Among the climatic variables, precipitation remained statistically stable over the analyzed period (*p* = 0.590). In contrast, a continuous and significant warming trend was observed, with mean annual temperature showing a consistent increase (*p* = 0.014). Indices reflecting water stress (TVDI) and surface moisture (LSWI) exhibited interannual variability associated with drought cycles, but no long-term directional trend was detected.

**Fig. 4:**
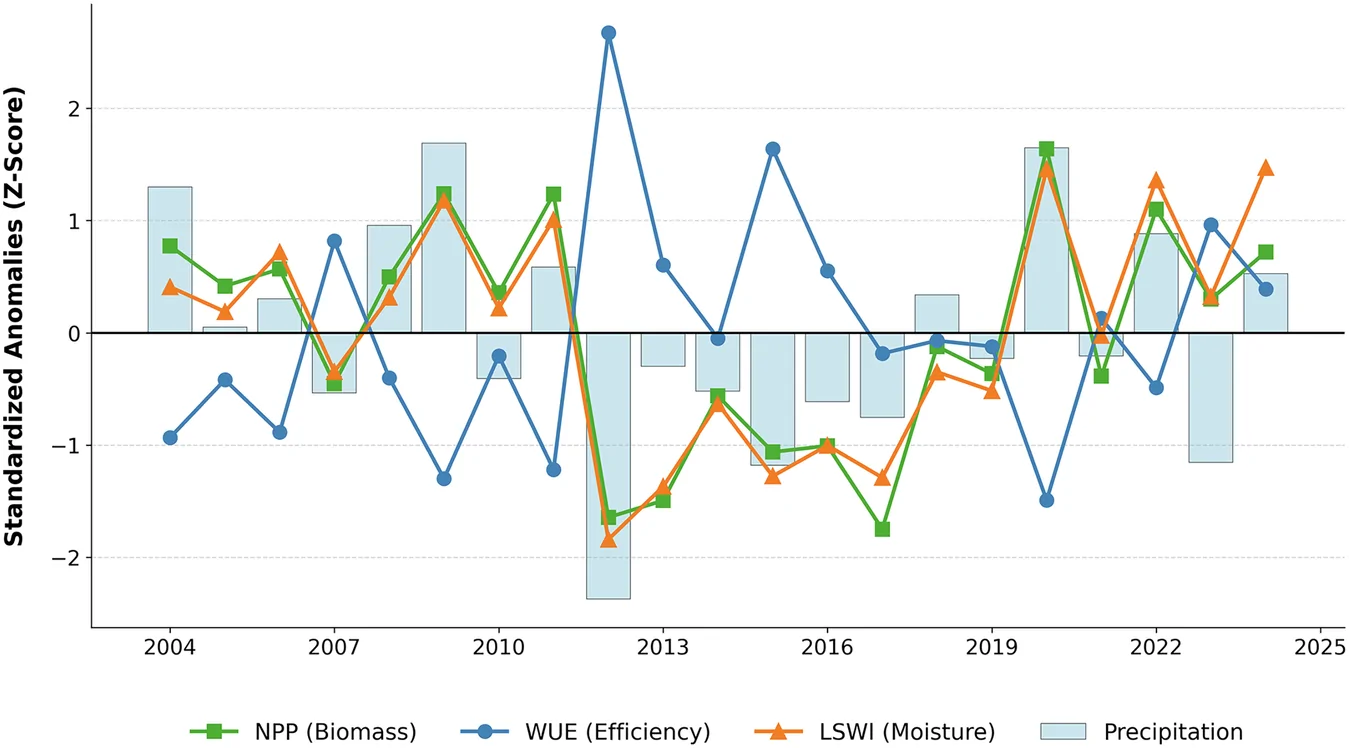
Ecosystem response mapping: productivity (NPP) versus efficiency (WUE) over time, in relation to environmental variables

A detailed examination of ecosystem response (Fig. 4) highlights a strong coupling of productivity (biomass) and water-use efficiency with surface moisture (LSWI) and precipitation pulses. Years marked by sharp precipitation deficits are immediately reflected in compensatory peaks or abrupt declines in WUE. This dynamic underscores the high water sensitivity of Caatinga vegetation and its capacity for rapid phenological adjustment. -+-+

### Drivers of Productivity Dynamics

**Fig. 5:**
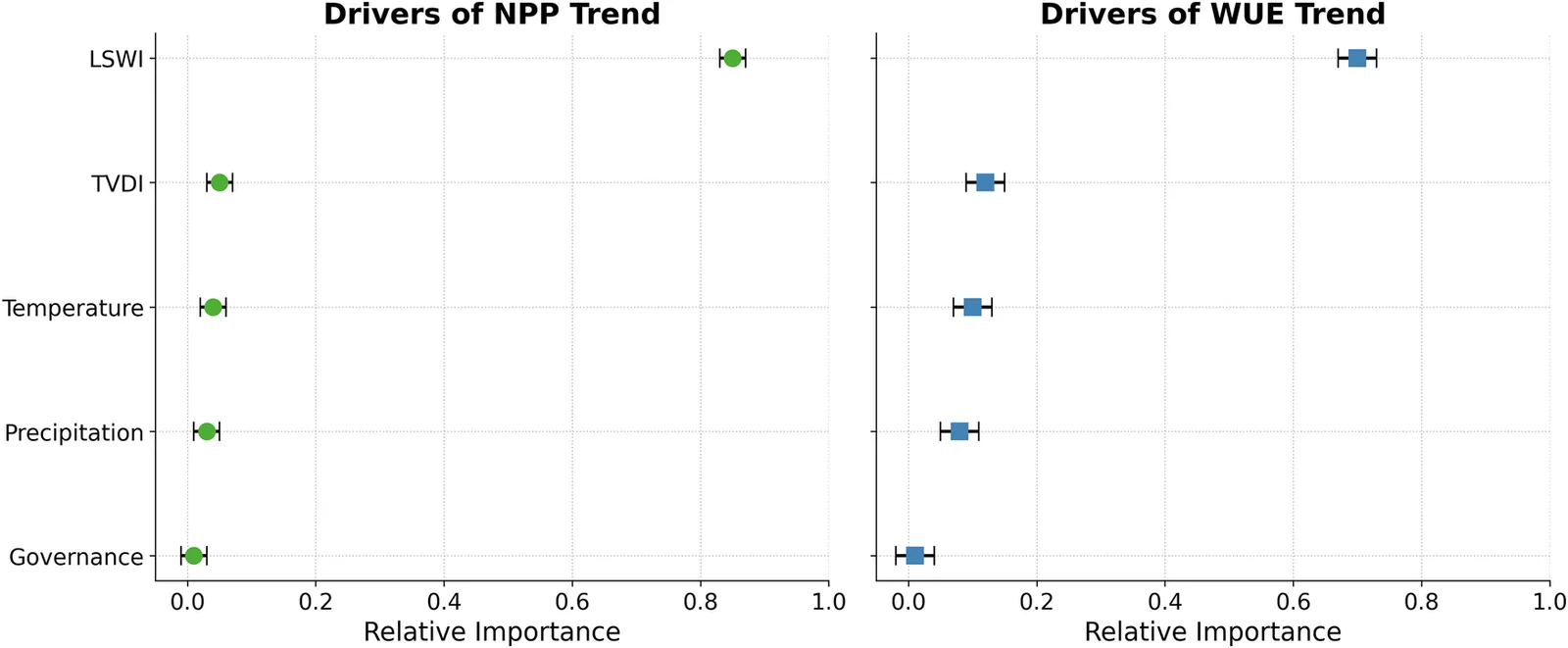
Relative importance of environmental variables and governance in driving NPP and WUE trends

Analysis of the environmental drivers influencing landscape dynamics (Fig. 5) confirmed that surface water availability, represented here by LSWI, is the dominant factor in the Caatinga biome. LSWI accounted for the majority of observed trends, with a relative importance of approximately 0.85 for NPP and 0.70 for WUE. This result highlights that water retention within the vegetation-soil system is more determinative than total rainfall; in other words, the system’s capacity to store water may be more important than the absolute amount of water entering the system.

In addition to LSWI, temperature and the drought index (TVDI) emerged as secondary but important drivers, particularly for WUE dynamics. Precipitation, when considered in isolation, showed lower explanatory power, suggesting that the spatiotemporal distribution of water plays a more critical role in phenology than total annual accumulation.

The results also indicate that governance regimes exert the lowest direct relative importance on strict interannual variation of these phenological metrics. This finding suggests that, at short interannual timescales, climatic factors act as the primary drivers of vegetation response, overriding the influence of land-use governance.

### The Role of Governance in Spatial Degradation, Restoration and Edge Effects

Although governance does not strongly influence interannual phenological variability, it acts as the primary structural determinant of long-term landscape conservation. Spatial analysis reveals a major challenge for territorial management in the biome: protected areas cover only a small fraction of the region. Considering the total extent of the Caatinga, Conservation Units (CUs) account for approximately 4.4 million hectares (5.09% of the biome). Traditional territories occupy even smaller areas, with Quilombola Territories covering 435.3 thousand hectares (0.50%) and Indigenous Lands (ILs) approximately 400.8 thousand hectares (0.46%).

**Fig. 6:**
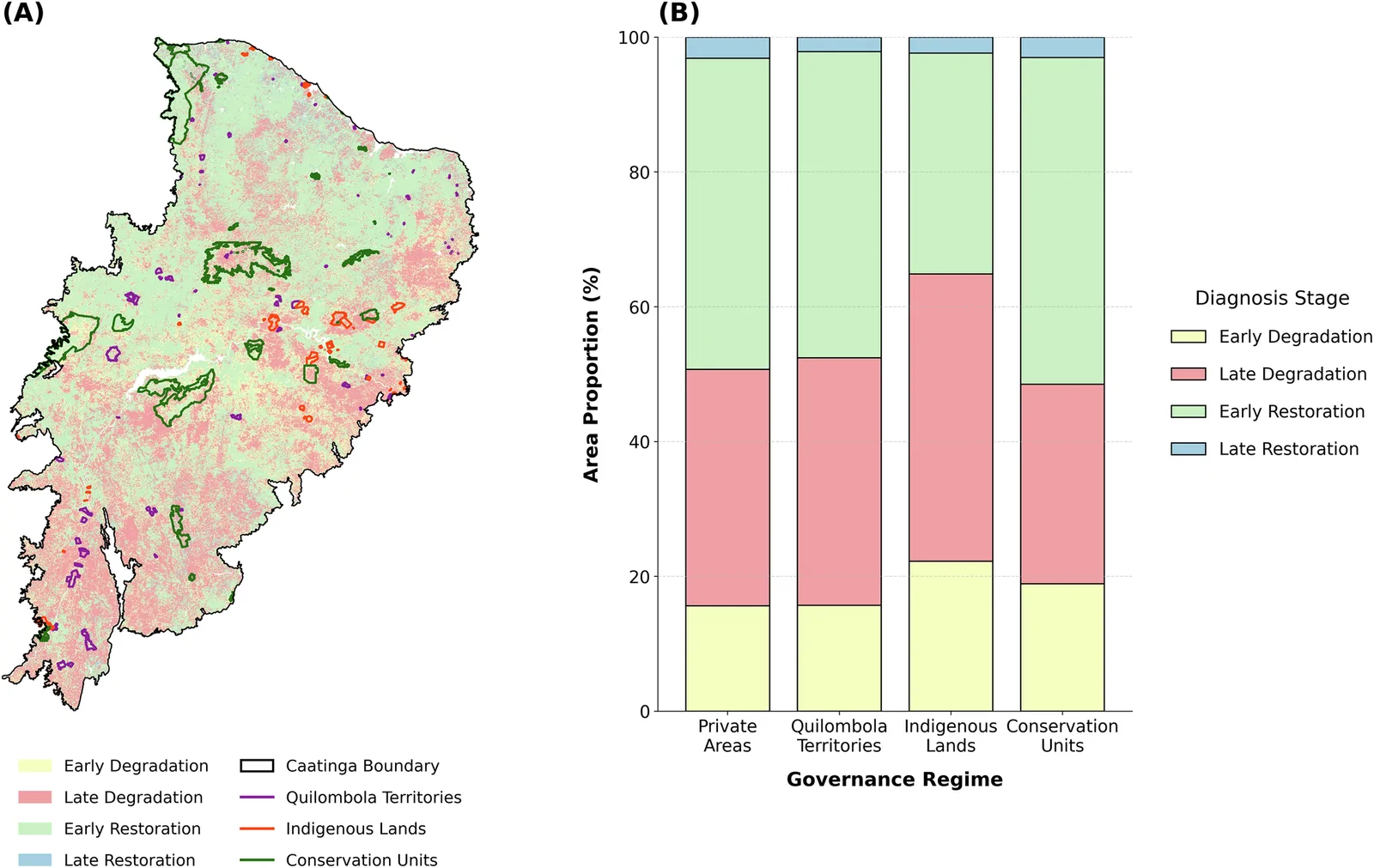
**A** Spatial distribution of degradation/restoration. **B** Proportion of area stratified by governance regimes

The spatial distribution of the assessment (Fig. 6A, B) indicates that Conservation Units function as the main ecological refugia in the region, concentrating the highest relative proportions of areas classified as “Early Restoration” and “Late Restoration”. In contrast, Indigenous Lands exhibit the highest internal proportion of degraded areas, followed by Quilombola Territories. In terms of absolute area, private properties concentrate the largest amount of areas undergoing restoration, which is expected given that they represent the overwhelming majority of the biome’s territory.

**Fig. 7:**
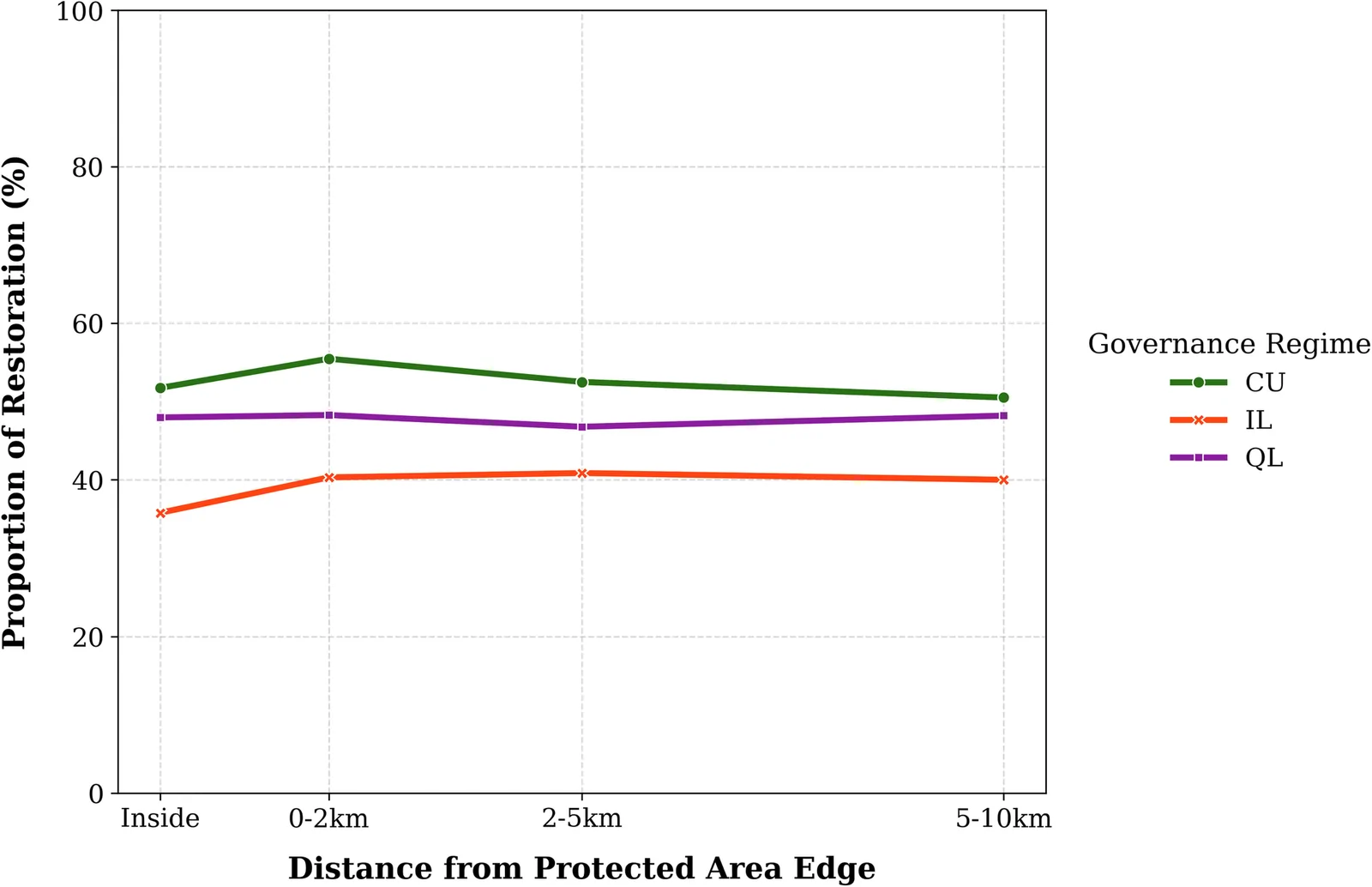
Edge effect analysis: percentage of restoration from the boundary to the core (up to 10 km)

Furthermore, the buffer analysis evaluating the spatial influence of these protected boundaries (Fig. 7) reveals distinct edge effects depending on the governance regime. Results show that Conservation Units maintain the highest levels of overall restoration, exhibiting a peak of stability and maximum protection within the first 2 km from the outer boundary, progressively dissipating as the distance towards the unprotected external matrix increases. Indigenous Lands and Quilombola Territories display slightly lower levels of overall restoration; however, they exhibit strong edge resilience. The proportion of restored areas within these territories remains nearly constant along the entire gradient, from the edge to the core (10 km).

## Discussion

### Ecosystem Resilience under Climate Warming

This study provides evidence that, despite significant regional warming and drying trends in South America and the Brazilian semiarid region (da Silva et al. 2025; Marengo et al. 2017; Rozante et al. 2025), the Caatinga has maintained stable levels of NPP and WUE. The capacity to maintain carbon balance under a scenario of increasing thermal pressure and frequent droughts suggests that the biome has not yet crossed critical functional tipping points.

These signs of resilience are particularly noteworthy given that the bioclimatic envelope of the Caatinga is rapidly shifting towards drier conditions. Recent projections indicate that future climate scenarios could reduce the equilibrium of aboveground biomass and expand arid areas over currently humid climates within the biome by the end of the century (Castanho et al. 2020; da Silva et al. 2023). Global drylands are prone to experiencing abrupt regime shifts when critical aridity thresholds are breached (Berdugo et al. 2022). The fact that the Caatinga still exhibits stable NPP trends implies a critical window of opportunity for environmental management and conservation efforts to prevent crossing these thresholds. However, the observed stability should not be interpreted as a sign of invulnerability, but rather as an indication that the system is currently operating within its adaptive capacity, which may be rapidly eroded if current trends persist or accelerate.

The persistence of productivity under these severe environmental conditions suggests that native species have developed highly specialized ecophysiological mechanisms. Recent eddy covariance studies corroborate our satellite-derived findings, demonstrating that the Caatinga exhibits a higher carbon use efficiency (CUE) compared to other dry forests worldwide, functioning as a highly efficient carbon sink (Mendes et al. 2025). Indeed, assessments of national greenhouse gas (GHG) inventories indicate that during specific recent years, carbon removals in the Caatinga represented nearly 50% of the total removals in Brazil (da Costa et al. 2025). This highlights the critical role of the Caatinga in national carbon dynamics and underscores the importance of maintaining its ecological integrity in the face of climate change.

However, this ecosystem-level resilience is not spatially uniform and is strongly mediated by land cover and structural characteristics. The maintenance of vegetation productivity during extreme drought events is heavily dependent on landscape complexity (de Araujo et al. 2022), where more complex and preserved landscapes effectively buffer the impacts of water deficits. Furthermore, local dynamics show that ecosystem WUE is strongly controlled by the duration of consecutive dry days rather than merely total precipitation amounts (Costa et al. 2022). This suggests that simplified or degraded landscapes are significantly more vulnerable to the ongoing warming trends.

From an environmental management perspective, these findings underscore that the Caatinga is not a barren wasteland, but a vital and active component of the global carbon cycle that requires immediate policy intervention. Given that macro-level adherence to climate treaties, such as the Paris Agreement, has the potential to halve the number of species at risk from climate change in Brazil (Malecha et al. 2025), local and regional governance should focus on maintaining the functional integrity of the biome. This includes not only the expansion of protected areas but also the promotion of sustainable land-use practices that enhance soil water retention and support the adaptive capacity of local vegetation.

### Dominance of Surface Moisture (LSWI) in Phenology

Analysis of environmental drivers indicates that surface moisture (LSWI) is the dominant predictor of phenological trends, surpassing aggregated annual precipitation. This finding suggests that vegetation response does not depend solely on total rainfall, but primarily on how water is infiltrated and retained within the soil root zone. In semiarid environments, rainfall events are highly concentrated and can generate high surface runoff, limiting effective soil water recharge (Silva et al. 2017). The strong coupling between LSWI and WUE indicates that monitoring leaf and soil moisture through remote sensing can provide a reliable representation of the ecological potential of the Caatinga.

Recent multifractal analyses of drylands demonstrate that water availability is the primary driver of vegetation dynamics, dictating the trade-offs between carbon gain and water-loss (Sanz et al. 2025; Sun et al. 2020). Maintaining high surface moisture allows the Caatinga vegetation to sustain its photosynthetic activities for longer periods, even during and after dry seasons. This capacity for water retention may be a key factor in the observed resilience of the biome, as it enables plants to maintain metabolic functions and carbon assimilation during periods of water scarcity.

Furthermore, the capacity to retain surface moisture is intrinsically linked to vegetation density and landscape preservation, where deforested and degraded areas exhibit significantly higher surface albedo and sensible heat fluxes, reflecting more solar radiation and altering the local microclimate (de Souza et al. 2023; Silva et al. 2024). In contrast, preserved or recovering dense Caatinga utilizes a larger fraction of net radiation for latent heat (evapotranspiration), which cools the environment and sustains higher Gross Primary Production (GPP) (de Oliveira et al. 2023). The structural complexity of the preserved canopy and understory may play a crucial role in dissipating the kinetic energy of rainfall, thereby reducing surface flow and enhancing soil moisture content (Andrade et al. 2020). This natural water management system is vital in a region where rising evapotranspiration rates and declining groundwater levels threaten regional water security (Almeida et al. 2024).

The observed strong coupling between productivity (NPP), water use efficiency (WUE), and surface moisture (LSWI) highlights the Caatinga’s unique ecohydrological dynamics compared to other seasonally dry ecosystems in Brazil, such as the Cerrado. While our results demonstrate that the Caatinga’s productivity is highly sensitive to short-term precipitation pulses and surface moisture retention, the Cerrado exhibits a distinct adaptive strategy. Evidence shows that in the Cerrado savanna, deep-rooted trees access deeper groundwater reserves, allowing them to sustain evapotranspiration and CO2 exchange even during prolonged dry seasons (da Rocha et al. 2002; Giambelluca et al. 2009). Consequently, canopy conductance and energy balance in the Cerrado are less constrained by immediate superficial soil moisture (Rodrigues et al. 2014). In contrast, the Caatinga’s predominant shrubby vegetation and shallower, rocky soils make its phenological responses more volatile and directly dependent on immediate water availability, placing these findings into a broader ecological context and justifying the dominance of LSWI as a primary driver of vegetation trajectories in our study.

These findings have direct implications for environmental management and restoration policies in the Brazilian semiarid region. Since vegetation productivity is highly dependent on surface moisture retention rather than precipitation alone, mitigation strategies should prioritize sustainable land-management practices—such as preventing overgrazing and promoting natural regeneration—that enhance soil water infiltration. Monitoring LSWI through remote sensing provides land managers with a robust, real-time indicator of ecohydrological resilience, enabling more targeted and effective interventions to combat desertification and support local livelihoods.

### Structural and Protective Role of Governance Regimes

While climatic forcings govern short-term interannual variability, the long-term spatial distribution of degradation and restoration is influenced by different forms of land governance. Over the last few decades, extensive land-cover mapping initiatives have shown that the Brazilian drylands experienced a continuous reduction in natural vegetation, primarily driven by the expansion of pasturelands and agriculture (Rocha et al. 2024; Souza et al. 2020). Against this backdrop of rapid landscape transformation, institutional and traditional governance regimes emerge as the primary structural determinants capable of halting extensive habitat conversion.

Beyond climatic drivers, the resilience of the Caatinga is profoundly shaped by land-use governance. As evidenced by our spatial and edge-effect results, Indigenous Lands and Quilombola Territories display remarkable resilience along the entire edge-to-core gradient. This spatial homogeneity suggests that land-use practices are spatially distributed in a sustainable and controlled manner by these traditional populations. The integration of local knowledge systems and customary institutions fosters conservation-oriented practices that act as vital barriers against external degradation, effectively buffering the ecosystem against chronic anthropogenic disturbances.

However, the absence of robust institutional mechanisms and coordinated public policies exacerbates the vulnerability of both the ecosystem and its inhabitants. Traditional approaches to drought mitigation in the Brazilian semi-arid region have heavily relied on reactive emergency subsidies. To safeguard the structural integrity of the Caatinga and prevent further desertification in the unprotected matrix, public policies must transition towards proactive landscape management. Effective governance must embrace a multi-level approach that legally empowers Indigenous Peoples and local communities, and implements robust financial mechanisms, such as Payments for Ecosystem Services (PES), to incentivize private landowners to conserve native vegetation.

In the mosaic of land use in the Caatinga, Conservation Units function as vital ecological refugia; by restricting large-scale land clearing, these protected areas maintain higher levels of biomass and structural complexity. However, simply protecting fragments from total clearance may often be insufficient (Antongiovanni et al. 2020). Chronic anthropogenic disturbances—such as continuous wood extraction, selective logging, and overgrazing—can severely erode biodiversity within formally protected boundaries if management enforcement is weak. Therefore, the high restoration rates observed within these units underscore the necessity of active management and continuous monitoring rather than mere legal designation.

Beyond formal protected areas, Indigenous Lands showed an effective conservation, highlighting the critical, yet historically neglected, contribution of Indigenous Peoples and Local Communities (IPLCs) to ecosystem resilience. Indigenous stewardship involves complex, place-based relational values and traditional practices that inherently promote the sustainable use of dryland resources (Dawson et al. 2023). Instead of separating humans from nature, these communities integrate forest gardens and local institutions to prevent external encroachment, proving that peopled landscapes can achieve conservation outcomes that often surpass those of strictly exclusionary protected areas.

In the absence of robust governance mechanisms, uncoordinated land-use changes often shift responsibilities among different economic sectors, marginalizing local populations and prioritizing short-term agricultural yields over long-term sustainability (Faggin et al. 2017; Siegmund-Schultze, 2021). To counteract this trend outside public lands, decentralized governance strategies are gaining traction. For instance, the establishment of Private Reserves of Natural Heritage (RPPNs) has shown significant potential in fostering natural vegetation recovery across Brazilian biomes (Nascibem et al. 2023), offering a complementary pathway to buffer the impacts of agribusiness expansion.

These contrasting realities suggest that a paradigm shift in dryland conservation approaches is needed. Establishing new strict protected areas without addressing the underlying socio-economic drivers and tenure rights often triggers environmental injustice and social conflicts. Effective governance in the Caatinga must embrace a multi-level approach that legally empowers IPLCs, integrates private landowners through incentives like Payments for Ecosystem Services, and enforces sustainable forest management practices that resonate with local needs rather than purely techno-bureaucratic models.

### Heterogeneity and Challenges in Quilombola Territories

Quilombola Territories exhibit a marked spatial dichotomy: they dominate the ranking of areas with the highest conservation success, while also appearing among territories in critical states of degradation. This polarized pattern suggests that the legal status of “Quilombo” encompasses contrasting land-tenure realities, socio-economic pressures, and levels of state support across the Caatinga biome.

This variation can be interpreted from the perspective of land tenure security. In communities where land rights are formally recognized, traditional practices appear to promote restoration in a stable and effective manner, suggesting that Afro-descendant lands in South America play a disproportionately positive role in mitigating forest loss and conserving irrecoverable carbon when their land rights are secure (Sangat et al. 2025).

However, in territories that remain unregularized, degradation may be accelerated due to the absence of clearly defined collective property rights (Baragwanath & Bayi, 2020). Without clearly defined and enforced collective property rights, these fragments become highly susceptible to chronic anthropogenic disturbances. Activities such as continuous wood extraction for firewood and charcoal, uncontrolled fire, and overgrazing gradually erode biodiversity and ecosystem biomass even in the absence of clear-cut deforestation (Antongiovanni et al. 2020; Faggin et al. 2017).

Therefore, advancing land tenure regularization for Afro-descendant communities is not merely a matter of historical reparation and social justice, but a critical environmental management imperative. To reverse the degradation trajectories in the most vulnerable territories, national environmental institutions must actively include these communities in decision-making processes, ensuring that conservation policies are coupled with targeted socio-economic support and secure land rights (Sangat et al. 2025).

### Edge Effects and Buffer Zones

The peak in restoration stability observed within the first 2 km of Conservation Units suggests that institutional boundaries may help absorb external pressures and, to some extent, limit the expansion of deforestation and elevated vapor pressure deficits. Rather than experiencing immediate degradation, these well-managed edges function as active buffer zones that shield the core areas from the expanding agricultural matrix, successfully limiting the encroachment of deforestation.

In contrast to humid tropical forests, where edge effects can strongly alter the internal microclimate conditions (Laurance et al. 2002), Caatinga vegetation appears to be well adapted to high solar radiation. Recent global assessments highlight that maintaining forest canopy cover is crucial for buffering extreme macroclimatic temperatures and creating microrefugia under climate change (Lombaerde et al. 2022).

Consequently, the primary drivers of edge degradation in this semiarid biome are not strictly microclimatic, but rather direct chronic anthropogenic disturbances. Activities such as selective logging, firewood extraction, and continuous livestock grazing frequently penetrate the unprotected borders of dry forest fragments (Antongiovanni et al. 2018, 2020). The notable stability observed deeper within Indigenous Lands and Quilombola Territories indicates that when local communities actively manage these borders and regulate resource extraction, the detrimental edge effects are significantly mitigated.

For environmental management and spatial planning, these findings emphasize that simply establishing isolated protected areas is insufficient to guarantee long-term ecosystem resilience. To safeguard the structural integrity of the Caatinga, policy frameworks must mandate the creation of managed buffer zones and promote sustainable agricultural practices in the surrounding matrix. Developing productive landscapes and green infrastructure around protected territories is essential to reduce edge contrast, minimize human intrusion, and establish functional biodiversity corridors across the semiarid region (Tabarelli et al. 2018).

### Implications for Environmental Management and Public Policy

Given the complex interplay between climate, ecohydrology, and land tenure, environmental management in the Caatinga strongly requires systemic and proactive policy frameworks. Traditional approaches to drought mitigation in the Brazilian semiarid region have heavily relied on reactive emergency subsidies. However, our findings suggest that public policies should transition towards fostering agricultural and land-management practices that enhance soil water retention at the landscape scale. Because formally protected areas cover only about 5% of the biome, ensuring regional resilience depends critically on engaging the private matrix. The implementation of robust financial mechanisms, such as Payments for Ecosystem Services (PES), is imperative to incentivize private landowners to conserve native vegetation and maintain the ecohydrological functions of the SVA system (da Silva et al. 2018; Siegmund-Schultze, 2021).

Furthermore, conservation strategies must move beyond the mere legal designation of protected areas. As shown by the spatial dichotomy in Quilombola Territories and the chronic degradation at unprotected edges, drawing boundaries on a map does not guarantee ecological integrity without underlying land tenure security. Efforts must prioritize urgent land regularization and targeted financial support for critically degraded traditional territories. National and regional governance models should actively include Indigenous Peoples and Afro-descendants in decision-making processes, shifting from top-down, techno-bureaucratic models to inclusive frameworks that empower local stewardship.

To support these complex management decisions, continuous and scalable monitoring systems are essential. Although medium-resolution data (e.g., 500 m) may introduce mixed-pixel effects at the microscale, the cloud-based processing of long-term satellite archives has proven highly effective for capturing systemic biome-level dynamics (Gorelick et al. 2017). Integrating ecophysiological variables (NPP and WUE) with surface moisture (LSWI) and dryness (TVDI) indices constitutes a robust early-warning framework. This approach allows decision-makers to decouple the effects of natural climate variability from human-induced degradation, identifying vulnerable hotspots before they cross irreversible desertification thresholds.

Ultimately, the path toward sustainability in the Caatinga dry forest requires a robust connection between the improvement of human livelihoods and the conservation of natural landscapes. By combining advanced remote sensing technologies with socially just governance and economic incentives, it is possible to design macro-policies and ecological zoning plans that not only mitigate climate change impacts but also secure the environmental and social future of the world’s most populous semiarid region.

## Conclusion

This study shows that Net Primary Productivity (NPP) and Water Use Efficiency (WUE) in the Caatinga remained resilient and stable over the past two decades (2004–2024), despite a significant regional warming trend. The interannual dynamics of these phenological variables are predominantly controlled by water availability (LSWI), surpassing the direct effect of precipitation alone. This suggests that local water retention capacity may serve as a key indicator of ecosystem health in the Caatinga.

While climate governs short-term interannual variability, the long-term trajectory of degradation and restoration is also influenced by land use, particularly by different governance regimes within the biome. Conservation Units appear to function as effective landscape buffers, concentrating higher restoration rates and showing resilience to edge effects. In contrast, Indigenous Lands showed greater vulnerability, concentrating high proportions of degraded areas, while Quilombola Territories exhibit a marked dichotomy, simultaneously ranking among areas with high conservation success and those experiencing severe degradation.

From an environmental planning and policy perspective, these findings suggest that the legal designation of protected areas alone may not ensure ecological integrity in the absence of land tenure security and institutional support. The expansion of protected areas should be accompanied by land tenure regularization strategies, particularly in vulnerable traditional territories, as well as economic incentives such as Payments for Ecosystem Services (PES), with a focus on non-protected areas that currently concentrate degradation processes in the Brazilian semiarid region.

## Supplementary information

Supplementary Information

## Data Availability

The remote sensing datasets used in this study are publicly available through the Google Earth Engine platform. Derived raster data and processed datasets are available from the corresponding author upon reasonable request.

## Appendix A Biophysical Modeling Equations

This section details the mathematical formulations used to calculate the individual components of the Fraction of Photosynthetically Active Radiation (FPAR), as well as the thermal and water stress coefficients for the optimized CASA model.

The individual components of FPAR (*F**P**A**R*_*N**D**V**I*_ and *F**P**A**R*_*S**R*_) were calculated assuming a linear relationship with the spectral indices, scaled using the 5th (*i*_*m**i**n*_) and 95th (*i*_*m**a**x*_) percentiles of the historical distribution for each land-cover class:

A1 $$\begin{array}{l}FPA{R}_{NDVI}=\frac{(NDVI-NDV{I}_{i,min})}{(NDV{I}_{i,max}-NDV{I}_{i,min})}\\ \hspace{0.5em}\times (FPA{R}_{max}-FPA{R}_{min})+FPA{R}_{min}\end{array}$$

A2 $$\begin{array}{l}FPA{R}_{SR}=\frac{(SR-S{R}_{i,min})}{(S{R}_{i,max}-S{R}_{i,min})}\\ \hspace{0.5em}\times (FPA{R}_{max}-FPA{R}_{min})+FPA{R}_{min}\end{array}$$

where *F**P**A**R*_*m**a**x*_ = 0.95 and *F**P**A**R*_*m**i**n*_ = 0.001.

The temperature stress coefficients (*T*_1_ and *T*_2_) reflect the vegetation’s response to ambient thermal conditions:

A3 $${T}_{1}(x,t)=0.8+0.02\times {T}_{opt}(x)-0.0005\times {({T}_{opt}(x))}^{2}$$

A4 $$\begin{array}{l}{T}_{2}(x,t)=1.1814\cdot {\left[1+{e}^{0.2\cdot ({T}_{opt}(x)-10-T(x,t))}\right]}^{-1}\\ \hspace{0.5em}\cdot {\left[1+{e}^{0.3\cdot (-{T}_{opt}(x)-10+T(x,t))}\right]}^{-1}\end{array}$$

where *T*_*o**p**t*_(*x*) is the optimum temperature for plant growth at a given pixel.

To model water stress (*W*_*ε*_), we employed an approach based on evaporative demand, using actual evapotranspiration (ET) and potential evapotranspiration (PET):

A5 $${W}_{\varepsilon }(x,t)=0.5+\left(0.5\times \frac{ET(x,t)}{PET(x,t)}\right)$$

## Code availability

The code scripts generated during the current study are available from the corresponding author upon reasonable request.
