# Supplementary material for: Ecosystem Resilience and Land-Use Governance: Remote Sensing Insights for Environmental Management in a Tropical Dry Forest
Source: Environ Manage. 2026 Sep 24;76(10):328. doi: 10.1007/s00267-026-02629-4 (PMC13601177; doi:10.1007/s00267-026-02629-4)
Supplement: Supplementary file 1 [file 267_2026_2629_MOESM1_ESM.pdf]

Electronic Supplementary Material

Ecosystem Resilience and Land-Use Governance: Remote Sensing Insights for Environmental Management in a Tropical Dry Forest

Lucas Nascimento da Silva   Bartolomeu Israel de Souza

Supplementary Tables and Figures

Table S1: Description of orbital and climate data collections used (2004–2024).

**Parameter**	**Product/Sensor**	**Resolution**	**Source**
Vegetation Indices	MOD13A1 V6.1	500 m	NASA LP DAAC
Reflectance	MOD09A1 V6.1	500 m	NASA LP DAAC
Surface Temp. (LST)	MOD11A2 V6.1	1 km	NASA LP DAAC
Evapotranspiration	MOD16A2GF V6.1	500 m	NASA LP DAAC
Precipitation	CHIRPS Daily	0.05°	UCSB-CHG
Climate Variables	ERA5-Land	0.1°	ECMWF
Land Use	MapBiomas Col. 8.0	30 m	MapBiomas
LSWI*	Derived from MOD09A1	500 m	This study
TVDI**	Derived from MOD11A2/MOD13A1	1 km	This study

* **LSWI (Land Surface Water Index):** Derived from MODIS NIR and SWIR bands; used as a proxy for vegetation and soil liquid water content, sensitive to canopy moisture.

** **TVDI (Temperature-Vegetation Dryness Index):** Derived from the integration of MODIS Land Surface Temperature (LST) and NDVI; used as an empirical indicator of soil moisture and thermal stress based on the LST/NDVI feature space.

Table S2: Maximum Light Use Efficiency (ε_{max}) parameters used in the CASA model for land cover classes (IGBP).

**IGBP Code**	**Land Cover Class**	ε_{max} $(gC \cdot MJ^{-1})$
1	Evergreen Needleleaf Forest (ENF)	0.962
2	Evergreen Broadleaf Forest (EBF)	1.268
3	Deciduous Needleleaf Forest (DNF)	1.086
4	Deciduous Broadleaf Forest (DBF)	1.165
5	Mixed Forest	1.051
6	Closed Shrublands	1.281
7	Open Shrublands	0.841
8	Woody Savannas	1.239
9	Savannas	1.206
10	Grasslands	0.860
12	Croplands	1.044
14	Cropland/Natural Vegetation Mosaics	1.044

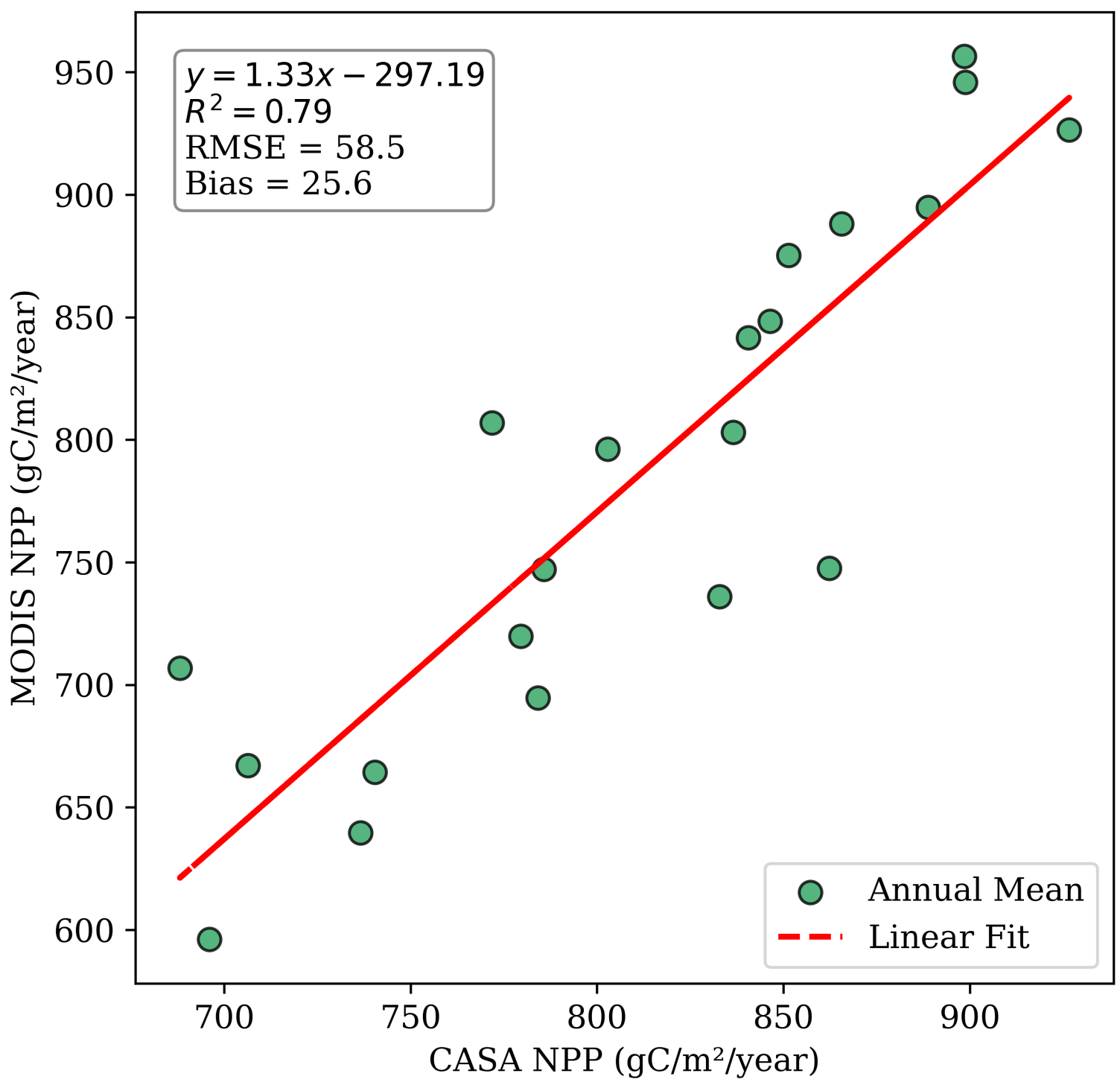

Figure S1: Cross-validation of the estimated CASA model versus MOD17A3HGF product.

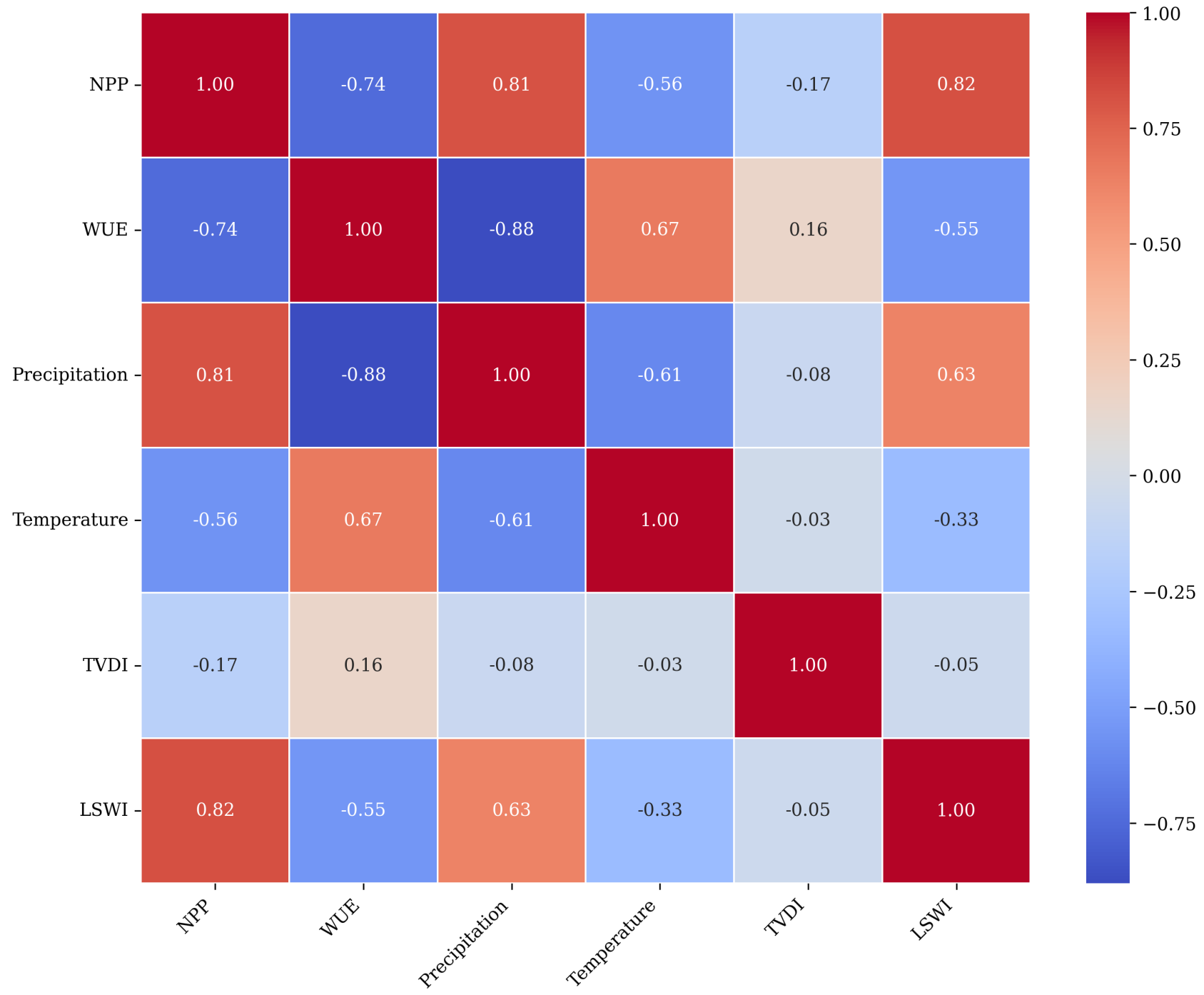

Figure S2: Correlation heatmap of interannual variations.

Table S3: Top 10 Most Conserved Protected Areas based on Restoration Status.

**Rank**	**Area Name**	**Governance Type**	**Total Area (ha)**	**Restoration (%)**
1	Jatobá	Quilombola Territory	197	100.00
2	Comunidade Aroeira	Quilombola Territory	567	100.00
3	Floresta Nac. de Sobral	Conservation Unit	591	100.00
4	Maacos	Quilombola Territory	961	100.00
5	Pitombeira	Quilombola Territory	345	100.00
6	Capoeiras	Quilombola Territory	887	100.00
7	Res. Extrativista Batoque	Conservation Unit	99	100.00
8	Engenho Bonfim	Quilombola Territory	74	100.00
9	Sabonete	Quilombola Territory	1,897	98.70
10	Brutos	Quilombola Territory	1,281	98.08

Table S4: Top 10 Most Degraded Protected Areas based on Degradation Status.

**Rank**	**Area Name**	**Governance Type**	**Total Area (ha)**	**Degradation (%)**
1	Vicentes	Quilombola Territory	345	100.00
2	Massacara	Indigenous Land	8,082	100.00
3	Karapotó	Indigenous Land	1,060	100.00
4	Catuabo	Quilombola Territory	887	100.00
5	Tuxá de Inajá	Indigenous Land	123	100.00
6	Kapinawá	Indigenous Land	12,444	98.42
7	Araça/Pato/Retiro	Quilombola Territory	19,244	94.75
8	Lagoa do Peixe	Quilombola Territory	6,530	94.34
9	Xukuru de Cimbres	Indigenous Land	1,158	93.62
10	Sítio Arruda	Quilombola Territory	370	93.33
